# Focus on Therapeutic Strategies of Nonalcoholic Fatty Liver Disease

**DOI:** 10.1155/2012/464706

**Published:** 2012-11-08

**Authors:** Marilena Durazzo, Paola Belci, Alessandro Collo, Enrica Grisoglio, Simona Bo

**Affiliations:** Department of Internal Medicine, University of Turin, 10127 Turin, Italy

## Abstract

Nonalcoholic fatty liver disease (NAFLD) is the most common chronic liver disease in the Western world (it affects 30% of the general adult population). The NAFLD encompasses a histological spectrum ranging from simple steatosis to nonalcoholic steatohepatitis (NASH), defined by steatosis, hepatocellular damage, and lobular inflammation in individuals without significant alcohol consumption and negative viral, congenital, and autoimmune liver disease markers. Currently, NAFLD is considered an emerging epidemic in light of the dramatic increase in obesity rates. With the progressive nature of NASH and its rising prevalence there is a significant need for a specific and targeted treatments since to date there has not been any validated therapies for NAFLD other than weight loss, which is well known to have a poor long-term success rate. In recent years, visceral adipose tissue has taken an important role in NAFLD pathogenesis, and current therapeutic approaches aim at reducing visceral obesity and free fatty acid overflow to the liver. This paper is focused on the treatments used for NAFLD and the potential new therapy.

## 1. Introduction

Nonalcoholic fatty liver disease (NAFLD) is the most common chronic liver disease in the Western world (it affects 30% of the general adult population) [1]. 

The NAFLD is an umbrella term for a group of diseases defined by a hepatic fat infiltration >5% hepatocyte, in the absence of excessive alcohol intake, defined by two standard drinks (20 g ethanol) daily for men and one standard drink (10 g ethanol) daily for women.

The NAFLD encompasses a histological spectrum ranging from simple steatosis to nonalcoholic steatohepatitis (NASH), defined by steatosis, hepatocellular damage, and lobular inflammation [2] in individuals without significant alcohol consumption and negative viral, congenital, and autoimmune liver disease markers. 

While steatosis does not carry the risk of progressive liver disease, patients with NASH are at risk of developing cirrhosis (20–30% of patients) [3].

NASH may progress to decompensated liver disease and result in liver failure.

Furthermore, NASH confers an increased risk of cardiovascular disease (CVD) and diabetes [4] both directly and through its association with other cardiometabolic abnormalities, including obesity and metabolic syndrome [5].

Currently NAFLD is considered an emerging epidemic in light of the dramatic increase in obesity rates. With the progressive nature of NASH and its rising prevalence, there is a significant need for a specific and targeted treatments since to date there has not been any validated therapies for NAFLD other than weight loss, which is well known to have a poor long-term success rate.

This paper is focused on the treatments used for NAFLD and the potential new therapy. Computerized advanced search for primary evidence was performed in PubMed (Public/Publisher MEDLINE) by using a combination of terminology and methodology search filters [6].

### 1.1. Pathogenesis: The Two-Hit Hypothesis

Currently the pathogenesis of NAFLD is unclear. NAFLD seems to be a multifactorial disease, combining both genetic and environmental factors. Several theories have been proposed and the “two-hit hypothesis” is the most accredited theory.

**Figure 1:**
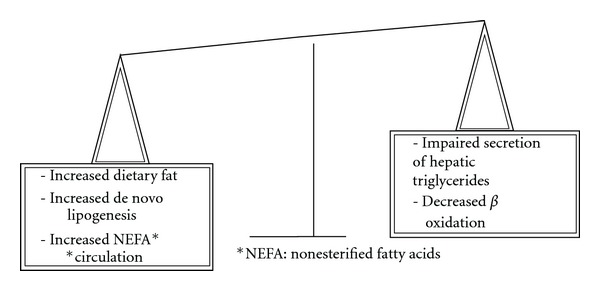
Pathways contributing to steatosis. An imbalance between fatty acid uptake, *de novo* synthesis and elimination of free fatty acids through oxidation and secretion into the blood with very low density lipoprotein triglycerides (VLDL), contributes to the development of steatosis.

Increased *de novo *lipogenesis [7], impaired secretion of hepatic triglyceride [8], decreased *β*-oxidation [9], and increased circulation of nonsterified fatty acids (NEFAs) released from adipose tissue [10] contribute to the steatosis when there is an imbalance between fatty acid uptake, *de novo* synthesis, and elimination of free fatty acids through oxidation and resecretion into the blood within very low density lipoprotein triglycerides (VLDL) (Figure 1). 

Steatosis represent the “first hit.” This increases the vulnerability of the liver to oxidative stress and inflammatory insults (the “second hit”) as hepatic lipid peroxidation [11], mitochondrial dysfunction [12], and inflammatory cells activation [13], which cause hepatocyte injury and the possible progression to NASH and cirrhosis.

The variable progression of NAFLD may be linked, in some patients, to genetic or environmental susceptibility that leads to hepatic fibrosis and ultimately cirrhosis [14]. 

According to new research on obese mice, the theory on the development of the NAFLD has been challenged. The same event can be the cause of fat infiltration, necroinflammation, and fibrosis; in this context the hepatic triglycerides (TG) accumulation may protect the hepatocyte from toxic free fatty acids (FFAs) improving hepatic steatosis but exacerbating liver injury and fibrosis [15].

Furthermore, adipokines and cytokines produced by adipose tissue play an important role in the pathogenesis of NAFLD. Some adipokines such as adiponectin and leptin may positively influence NAFLD while others, such as TNF-*α* and resistin, may negatively influence it [16]. Also insulin resistance (IR) seems to play a major role in the development of NAFLD in the accumulation of fat in the liver to progression in NASH [16]. Dysregulation of adipokines and cytokines is involved in the development of IR, fatty liver, and its progression to NASH [17].

## 2. Diagnosis 

Diagnosis of NAFLD is difficult because a completely reliable test to distinguish alcoholic and nonalcoholic fatty liver disease has not yet been found. Furthermore for the nature of NAFLD is mandatory to identify patients with progressive liver disease who are at risk of end-stage liver disease [18].

The diagnostic gold standard is *liver biopsy *(LB) but it is invasive, risky (complications like haemorrhage), costly and suffer for sampling [19].

The NASH Clinical Research Network designed and validated an histological score system (NAFLD activity score-NAS) to define the spectrum of NAFLD [20]; however, this score is not completely clear and it may incur in errors. So we need to find an alternative, not invasive exam for the diagnosis of this disorder and some exams to define which really need a LB. Biochemical and radiological methods are under development.

*Transient elastography* (Fibroscan), which measures liver stiffness (index of liver disease staging), accurately predict hepatic fibrosis, in a variety of clinical conditions like NASH, alcoholic hepatitis, viral hepatitis, and autoimmune liver disease [21–26], but Fibroscan has some limitations like the rate of unsuccessful examination in patients with metabolic syndrome because it has limited accuracy in the presence of obesity and steatosis [27, 28].

A method to evaluate advanced fibrosis is NAFLD fibrosis score. It is easy to calculate from routine parameters (age, hyperglycaemia, BMI, platelet count, albumin, and AST/ALT ratio) and has been independently validated in populations of various ethnicities, BMI, and diabetic status [29].

The NAFLD fibrosis score facilitates the identification of NAFLD patients with more advanced disease who require ongoing followup, and considerably reduces the requirement for liver biopsy in the minority of patients with an indeterminate score (25%) [30]. 

Nevertheless, the test is more useful to predict the absence of advanced fibrosis than its presence because its sensitivity is low and the specificity and the negative predictive value are high [31]. Several biomarkers are being investigated to differentiate between NASH and simple steatosis. *Adipocyte fatty acid binding protein* (AFABP) has a role in interaction between adipocytes and macrophages which leads to inflammation and insulin resistance [32].

Values of *blood cytokeratin 18 fragment* (CK-18) are linked with the degree of hepatocellular apoptosis, a character of NASH [33].

*Fibroblast growth factor 21* (FGF21) serum levels are high in NAFLD patients and its expression in the liver increases with the steatosis grade [34].

These results make biomarkers promising which could be used as noninvasive tests for NASH but they have not been adequately evaluated in independent cohorts, necessitating further research in this field.

A recent work showed that in patients with NAFLD all three biomarkers were associated with lobular inflammation, and CK-18 is the most accurate biomarker for NAFLD and NASH. Furthermore, a two-step approach using CK-18 and FGF21 improves the accuracy in diagnosing NASH [35].

Recent studies have underlined the role of non-HDL-cholesterol (non-HDL-C) as a superior predictor of cardiovascular incident than LDL-cholesterol [36].

Furthermore, recent study has shown that non-HDL-C levels increased more in patients with NASH than in those with steatosis, defining this value as a possible biomarker to estimate a patient's risk of NASH and need for a LB [37].

**Figure 2:**
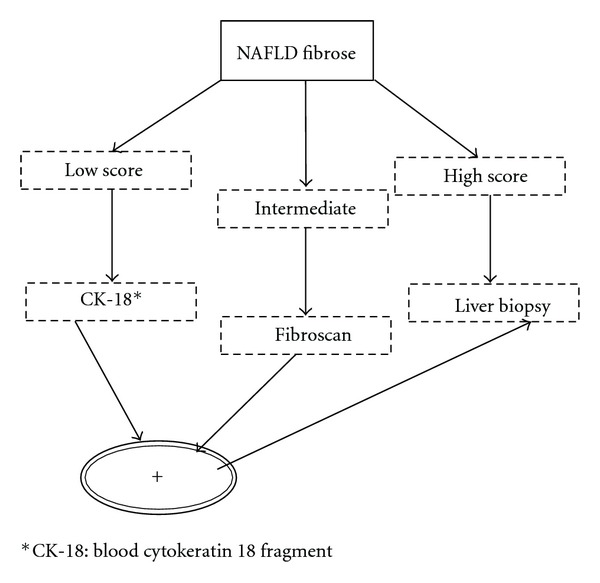
Possible algorithm for the diagnosis of NAFLD. *Limits*: fibroscan is possible in BMI <30 kg/m^2^. Adapted by Musso et al. [29].

More studies are needed to confirm this finding (Figure 2).

## 3. Therapy

In recent years visceral adipose tissue has taken an important role in NAFLD pathogenesis, and current therapeutic approaches aim at reducing visceral obesity and free fatty acid overflow to the liver (Table 1).

### 3.1. Lifestyle Interventions

Lifestyle interventions (diet and exercise) are the standard treatment of NAFLD. The purpose of lifestyle modification is weight loss; moreover, it has an important role in the decrease of multiple cardiometabolic risk factors.

A weight loss ≥5% improved steatosis and cardiometabolic risk factors, while a weight loss ≥7% improved necroinflammation and NAS [38, 39]. 

The effects of weight loss are inconstant for the limited compliance of patients to the diet and the exercise [40].

The correct composition of the diet for NAFLD is unknown but the importance of the diet in this disease is underlined by the improvement of insulin sensitivity, the reduction of hepatic FFAs supply, and adipose tissue inflammation [41].

Different studies show that caloric restriction is the most important goal for improving hepatic steatosis, but a different nutrient composition may carry additional benefits according to individual patients features. Recent studies are suggested that cholesterol, trans fat, and excessive fructose had a role in the pathogenesis of NAFLD [42].

Regular exercise has well-known benefits on metabolic abnormalities. Recent studies suggest aerobic exercise may reduce hepatic steatosis through hepatic adenosine monophosphate-activated protein kinase (AMPK) activation. This evidence could define the exercise's benefits on NAFLD not only for the weight loss [43].

### 3.2. Lipid-Lowering Drugs: Fibrates, Statins, *ω*-3 Polyunsaterated Fatty Acids (PUFA)

In the literature, there are some trials about the potential therapeutic role of lipid-lowering drugs in NAFLD. It seems that *fibrates* improve serum transaminases and may have a positive impact on IR and NAFLD [44]; *statins* are safe [45], improve serum levels of transaminases, and show beneficial effects on necroinflammation [46, 47]. Finally, *PUFA* improves biochemical and ultrasonographic steatosis [48]*.*

Further studies are needed to elucidate this issue.

### 3.3. Insulin Sensitizers: Metformin and Thiazolidinediones (TZDs)

Some works analysed the efficacy of* metformin* in NAFLD [49, 50]. Metformin reduced hepatic expression of TNF-*α*, a mediator of hepatic insulin resistance and necroinflammation, increased FFAs oxidation, and suppressed lipogenesis through AMPK activation [51]. Metformin had positive metabolic effects, improved weight loss and levels of liver enzymes [52].

The association between metformin and lifestyle intervention has given promising results, on the contrary there were controversial results on the improvement of histological steatosis, necroinflammation, and fibrosis [49, 53], necessitating further studies.

Few works have been studied on the role of *TZDs *pioglitazone and rosiglitazone in the treatment of NAFLD. They improved steatosis and necroinflammation, but their role on fibrosis had discordant results and is still not clear; however, TZDs significantly reduced the risk of fibrosis progression [54–56]. 

For the paucity of drugs used in the management of NAFLD, in recent years authors are investigating new therapeutic strategies.

### 3.4. Endocannabinoid Receptor Antagonists

The endocannabinoid pathway plays a significant role in the regulation of appetite and body weight, hepatic lipid metabolism and fibrosis. The endocannabinoid receptors-1 (CB-1) are overexpressed in NASH, suggesting a direct involvement of endocannabinoid system in the regulation of hepatocyte metabolism [57].

*Rimonabant*, a CB-1 blocker, in obese rats with NASH led to the improvement in lipid profile and the reduction in hepatic steatosis and fibrogenesis [58]. Depression and anxiety were more common with rimonabant. Concern about psychiatric adverse effects led to withdrawal of rimonabant, [59] but the development of peripherally acting CB1 antagonists is an area of intense research.

### 3.5. Orlistat

Orlistatis an inhibitor of gastric and pancreatic lipase. It inhibits the absorption of dietary triglycerides and has a role in reducing weight and IR in obesity. It could be used in patients with NAFLD for weight loss improvement.

The group of *Zelber-Sagi *have not noticed a difference of weight reduction between the orlistat and placebo groups. Therefore, other mechanisms of its beneficial effects in patients with NAFLD should be considered as the improvement of insulin sensitivity and the reduction of free fatty acids levels [60]. 

### 3.6. Angiotensin II Type 1 Receptor Blockers (ARBs)

The association between the angiotensin II type 1 receptor polymorphisms and the presence and severity of NAFLD [61] could be the base of evaluation of ARBs for reducing hepatic lipid accumulation in these patients [62].

### 3.7. Antioxidant Agents

The oxidative stress seems to have a role in the second hit of NAFLD. Several works have studied the effects of vitamins C and E in patients with NAFLD with the result of biochemical improvement (decrease of transaminases levels) and discordant results about histological improvement [63, 64].

These data are so limited to give any significance in the therapeutic strategies of NAFLD.

### 3.8. Ursodeoxycholic Acid (UDCA)

UDCA also has a role of antioxidant, improving liver enzymes but there are not enough data to indicate its use in NAFLD [65].

### 3.9. TNF-*α* Inhibitor

Anti-TNF therapy is based on the role of TNF-*α* in necroinflammation and insulin resistance.* Pentoxifylline* (a TNF-*α* inhibitor) in several trials improved liver enzymes and cytokine-mediated systemic inflammation [66] and reduced histological steatosis and necroinflammationin in patients with NASH [63] as demonstrated in the work of Zein at al. [67].

Further studies are required to confirm these results. 

### 3.10. Probiotics

Gut microbiota is linked to NAFLD by its proinflammatory metabolites through endotoxin-mediated toll-like receptor-4 axis activation [68] and may trigger hepatic *de novo* lipogenesis enzymes [69]; moreover, small intestinal overgrowth is plausibly linked to NAFLD pathogenesis [70]. Prebiotics are defined as “a nondigestible food ingredient that beneficially affects the host, by selectively stimulating the growth and/or activity of one or a limited number of bacteria in the colon.” Prebiotic fibres alter the gut microbiota in a manner that is advantageous to the host [71].

Animal models have shown that prebiotics reduces plasma lipid and hepatic triglyceride concentration and have a cholesterol-lowering effect, also prebiotic-rich diet may ameliorate NAFLD by attenuating *de novo *fatty acid synthesis [72, 73].

To date, continued ingestion of prebiotics is necessary to maintain these changes and the minimum dose and the duration of the therapy has not been defined, necessitating further studies [74].

In the literature, there are few studies on the effects of prebiotic on NAFLD patients, which showed a decrease in cholesterol and triglycerides levels, but the reduction is lower than in animal models, probably for the relatively lower dose administered [75] and for the lower rate of the hepatic *de novo* lipogenesis in humans [76].

Further studies in humans are required to understand better the potential to translate the positive effects of prebiotic fibres noted in animal models to human clinical application.

### 3.11. Green Tea Extract (GTE)

Catechins are the major polyphenols present in green tea and have antioxidant, antinflammatory, and enzyme inhibition activities [77]. GTE protects against hepatic steatosis and injury in the *ob/ob* mice model of NAFLD [78] through several activities which include decreasing absorption of dietary lipid and lipogenic substrates [79], suppressing adipose lipolysis and decreasing hepatic and adipose lipogenesis [80]. GTE also increases energy expenditure by upregulating *β*-oxidation and thermogenesis responses [81] and improving insulin sensitivity [82].

GTE also has direct and indirect antioxidant and anti-inflammatory properties that could prevent the progression from steatosis to NASH by directly scavenging reactive oxygen and nitrogen species, by upregulating the transcription of genes related to the cellular antioxidant defense, and by directly suppressing inflammatory responses [83].

To date, no RTS in humans with NAFLD have examined the potentially beneficial effects of green tea. Such studies are necessary to provide direct evidence that green tea could have a role in the reduction of development and/or progression of NAFLD in humans. 

### 3.12. Incretin Analogs/Antagonists

Incretin analogs and antagonists are a new class of drugs for the treatment of diabetes. They are dipeptidyl peptidase-4 (DPP-4) inhibitors, glucagon-like peptide-1 (GLP-1) analogs and glucose-dependent insulinotropic polypeptide (GIP) agonists. Their action stimulates pancreatic *β*-cell insulin secretion and growth.

The focus for the use of these medicaments in NAFLD is the demonstration of reduced incretin action in NASH patients [84].

In animal models GLP-1 analogs decrease hepatic lipogenesis and oxidative stress [85]. 

Studies on GIP action suggest that GIP may be an important mediator of the adypocite response to nutritional excess and may have a role in the metabolic risk of NAFLD [86].

### 3.13. Thyromimetics

Recent research on antiobesity and low density lipoprotein (LDL) cholesterol lowering effects of thyroid hormones have developed a class of drugs with these beneficial effects, such as thyromimetics [87]. 

Animal models showed steatosis improvement and reduction in hepatic lipoperoxidaton [88]. 

More research is needed in human trials.

### 3.14. Nuclear Transcription Factors: Pregnane X Receptor and Farnesoid X Receptor

Studies on mice have shown that pregnane X receptor (PXR) is implicated in hepatic steatosis and in lipid and glucose metabolism [89]. 

PXR has also been proposed as a potential target for antifibrotic therapy [90] but other studies are needed to confirm these features and to characterize synthetic PXR agonists. 

Farnesoid X receptor (FXR) is a regulator of lipid and glucose homeostasis. In the liver, FXR influences lipid homeostasis and antagonizes inflammatory and fibrogenetic process [91].

Several synthetic FXR agonists are studied for the therapy of metabolic and hepatic disorders [92].

### 3.15. Bariatric Surgery

A key point of NAFLD is exogenous fat accumulation which is reduced by surgery. Different surgical procedures, as laparoscopic adjustable banding, biliopancreatic diversion, vertically banded gastroplasty, and Roux-en y gastric bypass have given good results improving steatosis, hepatocellular injury, and fibrosis in NAFLD patients without signs of other liver injury [93], but there are no trials about the long-term effects of these procedures. 

For patients with NASH not responding to lifestyle intervention, pharmacological treatment should be considered.

## 4. Conclusion

NAFLD in these years has obtained a prominent role in the spectrum of liver diseases for the increased frequency and recognition. Also, in the absence of a target therapy, it may appear as the leading cause of cirrhosis and liver transplantation by 2020. To date the gold standard for the therapy of NAFLD is lifestyle intervention. A gradual weight loss is desirable, because a faster weight loss has exacerbated liver injury [94].

For patients not compliant to lifestyle intervention, there is not a validated regimen, the result of recent studies suggest that a combination therapy occur targeting different mechanisms involved in the pathogenesis of NAFLD [64, 95].

In the future another feature to study may be the association between lifestyle intervention and drugs, to assess if they could be a synergistic effect on liver histology [96].

## Figures and Tables

**Table 1:** Synthesis of possible targets of therapeutic strategies in use and in study for NAFLD. Focus on biochemical, ultrasonographic, and histological features of NAFLD: altered levels of transaminases, steatosis, necroinflammation, and fibrosis.

	Biochemical features	Steatosis	Inflammation	Fibrosis
Diet	+	+ (>5% weight loss)	+ (>7% weight loss)	
Exercise		+		
Fibrates	+			
Statins	+		+	
PUFA	+	+		
Metformin	+	±	±	±
TZDs		+	+	±
Rimonabant		+		+
Orlistat*				
ARBs*				
Antioxidant agents	+			
UDCA	+			
Pentoxifylline	+	+	+	
Probiotics*				
GTE		+	+	
Incretin anlogs/antagonists			+	
Thyromimetics		+		
PXR		+		+
FXR			+	+
Bariatric surgery		+	+	+

*Target not clear.

PUFA: ω-3 polyunsaterated fatty acids; TZDs: thiazolidinediones; ARBs: angiotensin II type 1 receptor blockers; UDCA: ursodeoxycholic acid; GTE: green tea extract; PXR: pregnane X receptor; FXR: farnesoid X receptor.

## References

[1] Musso G, Gambino R, Cassader M (2010). Non-alcoholic fatty liver disease from pathogenesis to management: an update. *Obesity Reviews*.

[2] Farrell GC, Hall P, George J, McCullough AJ (2005). *Fatty Liver Disease: NASH and Related Disorders*.

[3] Farrell GC, Larter CZ (2006). Nonalcoholic fatty liver disease: from steatosis to cirrhosis. *Hepatology*.

[4] Musso G, Gambino R, Cassader M, Pagano G (2011). Prognosis and non-invasive methods to assess liver disease severity in non-alcoholic fatty liver disease (NAFLD): systematic review and meta-analysis. *Annals of Medicine*.

[5] Ghouri N, Preiss D, Sattar N (2010). Liver enzymes, nonalcoholic fatty liver disease, and incident cardiovascular disease: a narrative review and clinical perspective of prospective data. *Hepatology*.

[6] Wilczynski NL, Haynes RB Robustness of empirical search strategies for clinical content in MEDLINE.

[7] Diraison F, Moulin PH, Beylot M (2003). Contribution of hepatic de novo lipogenesis and reesterification of plasma non esterified fatty acids to plasma triglyceride synthesis during non-alcoholic fatty liver disease. *Diabetes and Metabolism*.

[8] Charlton M, Sreekumar R, Rasmussen D, Lindor K, Nair KS (2002). Apolipoprotein synthesis in nonalcoholic steatohepatitis. *Hepatology*.

[9] Savage DB, Cheol SC, Samuel VT (2006). Reversal of diet-induced hepatic steatosis and hepatic insulin resistance by antisense oligonucleotide inhibitors of acetyl-CoA carboxylases 1 and 2. *Journal of Clinical Investigation*.

[10] Donnelly KL, Smith CI, Schwarzenberg SJ, Jessurun J, Boldt MD, Parks EJ (2005). Sources of fatty acids stored in liver and secreted via lipoproteins in patients with nonalcoholic fatty liver disease. *Journal of Clinical Investigation*.

[11] Chung MY, Yeung SF, Park HJ, Volek JS, Bruno RS (2010). Dietary *α*- and *γ*-tocopherol supplementation attenuates lipopolysaccharide-induced oxidative stress and inflammatory-related responses in an obese mouse model of nonalcoholic steatohepatitis. *Journal of Nutritional Biochemistry*.

[12] Pérez-Carreras M, Del Hoyo P, Martín MA (2003). Defective hepatic mitochondrial respiratory chain in patients with nonalcoholic steatohepatitis. *Hepatology*.

[13] Yang SQ, Lin HZ, Lane MD, Clemens M, Diehl AM (1997). Obesity increases sensitivity to endotoxin liver injury: implications for the pathogenesis of steatohepatitis. *Proceedings of the National Academy of Sciences of the United States of America*.

[14] Kim CH, Younossi ZM (2008). Nonalcoholic fatty liver disease: a manifestation of the metabolic syndrome. *Cleveland Clinic Journal of Medicine*.

[15] Yamaguchi K, Yang L, McCall S (2007). Inhibiting triglyceride synthesis improves hepatic steatosis but exacerbates liver damage and fibrosis in obese mice with nonalcoholic steatohepatitis. *Hepatology*.

[16] Polyzos SA, Kountouras J, Zavos C (2009). Nonalcoholic fatty liver disease: the pathogenetic roles of insulin resistance and adipocytokines. *Current Molecular Medicine*.

[17] Jarrar MH, Baranova A, Collantes R (2008). Adipokines and cytokines in non-alcoholic fatty liver disease. *Alimentary Pharmacology and Therapeutics*.

[18] Ekstedt M, Franzén LE, Mathiesen UL (2006). Long-term follow-up of patients with NAFLD and elevated liver enzymes. *Hepatology*.

[19] Ratziu V, Bugianesi E, Dixon J (2007). Histological progression of non-alcoholic fatty liver disease: a critical reassessment based on liver sampling variability. *Alimentary Pharmacology and Therapeutics*.

[20] Kleiner DE, Brunt EM, Van Natta M (2005). Design and validation of a histological scoring system for nonalcoholic fatty liver disease. *Hepatology*.

[21] Ziol M, Handra-Luca A, Kettaneh A (2005). Noninvasive assessment of liver fibrosis by measurement of stiffness in patients with chronic hepatitis C. *Hepatology*.

[22] Fraquelli M, Rigamonti C, Casazza G (2007). Reproducibility of transient elastography in the evaluation of liver fibrosis in patients with chronic liver disease. *Gut*.

[23] Marcellin P, Ziol M, Bedossa P (2009). Non-invasive assessment of liver fibrosis by stiffness measurement in patients with chronic hepatitis B. *Liver International*.

[24] Wong VWS, Vergniol J, Wong GLH (2010). Diagnosis of fibrosis and cirrhosis using liver stiffness measurement in nonalcoholic fatty liver disease. *Hepatology*.

[25] Nahon P, Kettaneh A, Tengher-Barna I (2008). Assessment of liver fibrosis using transient elastography in patients with alcoholic liver disease. *Journal of Hepatology*.

[26] Corpechot C, El Naggar A, Poujol-Robert A (2006). Assessment of biliary fibrosis by transient elastography in patients with PBC and PSC. *Hepatology*.

[27] Petta S, Di Marco V, Cammà C, Butera G, Cabibi D, Craxì A (2011). Reliability of liver stiffness measurement in non-alcoholic fatty liver disease: the effects of body mass index. *Alimentary Pharmacology and Therapeutics*.

[28] Vizzutti F, Arena U, Marra F, Pinzani M (2009). Elastography for the non-invasive assessment of liver disease: limitations and future developments. *Gut*.

[29] Musso G, Gambino R, Cassader, Pagano M (2011). Meta-analysis: natural history of non-alcoholic fatty liver disease (NAFLD) and diagnostic accuracyof non invasive tests for liver disease severity. *Annals of Medicine*.

[30] Angulo P, Hui JM, Marchesini G (2007). The NAFLD fibrosis score: a noninvasive system that identifies liver fibrosis in patients with NAFLD. *Hepatology*.

[31] McPherson S, Stewart SF, Henderson E, Burt AD, Day CP (2010). Simple non-invasive fibrosis scoring systems can reliably exclude advanced fibrosis in patients with non-alcoholic fatty liver disease. *Gut*.

[32] Milner KL, van der Poorten D, Xu A (2009). Adipocyte fatty acid binding protein levels relate to inflammation and fibrosis in nonalcoholic fatty liver disease. *Hepatology*.

[33] Feldstein AE, Wieckowska A, Lopez AR, Liu YC, Zein NN, McCullough AJ (2009). Cytokeratin-18 fragment levels as noninvasive biomarkers for nonalcoholic steatohepatitis: a multicenter validation study. *Hepatology*.

[34] Li H, Fang Q, Gao F (2010). Fibroblast growth factor 21 levels are increased in nonalcoholic fatty liver disease patients and are correlated with hepatic triglyceride. *Journal of Hepatology*.

[35] Shen J, Chan HL, Wong GL (2012). Non-invasive diagnosis of non-alcoholic steatohepatitis by combined serum biomarkers. *Journal of Hepatology*.

[36] Robinson JG, Wang S, Smith BJ, Jacobson TA (2009). Meta-analysis of the relationship between non-high-density lipoprotein cholesterol reduction and coronary heart disease risk. *Journal of the American College of Cardiology*.

[37] Corey KE, Lai M, Gelrud L (2012). Non-high-density lipoprotein cholesterol as a biomarker for nonalcoholic steatohepatitis. *Clinical Gastroenterology and Hepatology*.

[38] Hatzitolios A, Savopoulos C, Lazaraki G (2004). Efficacy of omega-3 fatty acids, atorvastatin and orlistat in non-alcoholic fatty liver disease with dyslipidemia. *Indian Journal of Gastroenterology*.

[39] Promrat K, Kleiner DE, Niemeire HM (2008). Randomized controlled trial testing the effects of weight loss on non-alcoholic steatohepatitis (NASH). *Hepatology*.

[40] Vuppalanchi R, Chalasani N (2009). Nonalcoholic fatty liver disease and nonalcoholic steatohepatitis: selected practical issues in their evaluation and management. *Hepatology*.

[41] Kashi MR, Torres DM, Harrison SA (2008). Current and emerging therapies in nonalcoholic fatty liver disease. *Seminars in Liver Disease*.

[42] Zelber-Sagi S, Nitzan-Kaluski D, Goldsmith R (2007). Long term nutritional intake and the risk for non-alcoholic fatty liver disease (NAFLD): a population based study. *Journal of Hepatology*.

[43] Musso G, Gambino R, Cassader M (2009). Recent insights into hepatic lipid metabolism in non-alcoholic fatty liver disease (NAFLD). *Progress in Lipid Research*.

[44] Basaranoglu M, Acbay O, Sonsuz A (1999). A controlled trial of gemfibrozil in the treatment of patients with nonalcoholic steatohepatitis. *Journal of Hepatology*.

[45] Chalasani N (2005). Statins and hepatotoxicity: focus on patients with fatty liver. *Hepatology*.

[46] Rallidis LS, Drakoulis CK, Parasi AS (2004). Pravastatin in patients with nonalcoholic steatohepatitis: results of a pilot study. *Atherosclerosis*.

[47] Hyogo H, Tazuma S, Arihiro K (2008). Efficacy of atorvastatin for the treatment of nonalcoholic steatohepatitis with dyslipidemia. *Metabolism*.

[48] Tanaka N, Sano K, Horiuchi A, Tanaka E, Kiyosawa K, Aoyama T (2008). Highly purified eicosapentaenoic acid treatment improves nonalcoholic steatohepatitis. *Journal of Clinical Gastroenterology*.

[49] Bugianesi E, Gentilcore E, Manini R (2005). A randomized controlled trial of metformin versus vitamin E or prescriptive diet in nonalcoholic fatty liver disease. *American Journal of Gastroenterology*.

[50] Haukeland JW, Konopski Z, Loberg EM (2008). A randomized placebo controlled trial with metformin in patients with NAFLD. *Hepatology*.

[51] Shoelson SE, Herrero L, Naaz A (2007). Obesity, inflammation, and insulin resistance. *Gastroenterology*.

[52] Mattar SG, Velcu LM, Rabinovitz M (2005). Surgically-induced weight loss significantly improves nonalcoholic fatty liver disease and the metabolic syndrome. *Annals of Surgery*.

[53] Loomba R, Lutchman G, Kleiner DE (2009). Clinical trial: pilot study of metformin for the treatment of non-alcoholic steatohepatitis. *Alimentary Pharmacology and Therapeutics*.

[54] Promrat K, Lutchman G, Uwaifo GI (2004). A pilot study of pioglitazone treatment for nonalcoholic steatohepatitis. *Hepatology*.

[55] Ratziu V, Giral P, Jacqueminet S (2008). Rosiglitazone for nonalcoholic steatohepatitis: one-year results of the randomized placebo-controlled Fatty Liver Improvement With Rosiglitazone Therapy (FLIRT) Trial. *Gastroenterology*.

[56] Aithal GP, Thomas JA, Kaye PV (2008). Randomized, placebo-controlled trial of pioglitazone in nondiabetic subjects with nonalcoholic steatohepatitis. *Gastroenterology*.

[57] Lichtman AH, Cravatt BF (2005). Food for thought: endocannabinoid modulation of lipogenesis. *Journal of Clinical Investigation*.

[58] Gary-Bobo M, Elachouri G, Gallas JF (2007). Rimonabant reduces obesity-associated hepatic steatosis and features of metabolic syndrome in obese zucker fa/fa rats. *Hepatology*.

[59] Food and Drug Administration Advisory Committee US (2007). *FDA Briefing Document: Zimulti (Rimonabant) Tablets, 20 mg*.

[60] Zelber-Sagi S, Kessler A, Brazowsky E (2006). A double-blind randomized placebo-controlled trial of orlistat for the treatment of nonalcoholic fatty liver disease. *Clinical Gastroenterology and Hepatology*.

[61] Yoneda M, Hotta K, Nozaki Y (2009). Association between angiotensin II type 1 receptor polymorphisms and the occurrence of nonalcoholic fatty liver disease. *Liver International*.

[62] Rosselli MS, Burgueño AL, Carabelli J, Schuman M, Pirola CJ, Sookoian S (2009). Losartan reduces liver expression of plasminogen activator inhibitor-1 (PAI-1) in a high fat-induced rat nonalcoholic fatty liver disease model. *Atherosclerosis*.

[63] Lirussi F, Azzalini L, Orando S, Orlando R, Angelico F (2007). Antioxidant supplements for non-alcoholic fatty liver disease and/or steatohepatitis. *Cochrane Database of Systematic Reviews*.

[64] Sanyal AJ (2009). A randomized controlled trial of pioglitazone or vitamin E for nonalcoholic steatohepatitis (PIVENS). *Hepatology*.

[65] Orlando R, Azzalini L, Orando S, Lirussi F (2007). Bile acids for non-alcoholic fatty liver disease and/or steatohepatitis. *Cochrane Database of Systematic Reviews*.

[66] Satapathy SK, Garg S, Chauhan R (2004). Beneficial effects of tumor necrosis factor-*α* inhibition by pentoxifylline on clinical, biochemical, and metabolic parameters of patients with nonalcoholic steatohepatitis. *American Journal of Gastroenterology*.

[67] Zein CO, Lopez R, Fu X (2012). Pentoxifylline decreases oxidized lipid products in nonalcoholic steatohepatitis: new evidence on the potential therapeutic mechanism. *Hepatology*.

[68] Malaguarnera M, Gargante MP, Russo C (2010). L-carnitine supplementation to diet: a new tool in treatment of nonalcoholic steatohepatitisa randomized and controlled clinical trial. *American Journal of Gastroenterology*.

[69] Musso G, Gambino R, Cassader M (2010). Gut microbiota as a regulator of energy homeostasis and ectopic fat deposition: mechanisms and implications for metabolic disorders. *Current Opinion in Lipidology*.

[70] Nardone G, Rocco A (2004). Probiotics: a potential target for the prevention and treatment of steatohepatitis. *Journal of Clinical Gastroenterology*.

[71] Roberfroid MB (2000). Prebiotics and probiotics: are they functional foods?. *American Journal of Clinical Nutrition*.

[72] Sugatani J, Wada T, Osabe M, Yamakawa K, Yoshinari K, Miwa M (2006). Dietary inulin alleviates hepatic steatosis and xenobiotics-induced liver injury in rats fed a high-fat and high-sucrose diet: association with the suppression of hepatic cytochrome P450 and hepatocyte nuclear factor 4*α* expression. *Drug Metabolism and Disposition*.

[73] Delzenne NM, Williams CM (2002). Prebiotics and lipid metabolism. *Current Opinion in Lipidology*.

[74] Parnell JA, Reimer RA (2010). Effect of prebiotic fibre supplementation on hepatic gene expression and serum lipids: a dose-response study in JCR:LA-cp rats. *British Journal of Nutrition*.

[75] Beylot M (2005). Effects of inulin-type fructans on lipid metabolism in man and in animal models. *British Journal of Nutrition*.

[76] Diraison F, Beylot M (1998). Role of human liver lipogenesis and reesterification in triglycerides secretion and in FFA reesterification. *American Journal of Physiology*.

[77] Lambert JD, Sang S, Yang CS (2007). Biotransformation of green tea polyphenols and the biological activities of those metabolites. *Molecular Pharmaceutics*.

[78] Bruno RS, Dugan CE, Smyth JA, DiNatale DA, Koo SI (2008). Green tea extract protects leptin-deficient, spontaneously obese mice from hepatic steatosis and injury. *Journal of Nutrition*.

[79] Juhel C, Armand M, Pafumi Y, Rosier C, Vandermander J, Lairon D (2000). Green tea extract (AR25) inhibits lipolysis of triglycerides in gastric and duodenal medium in vitro. *Journal of Nutritional Biochemistry*.

[80] Shrestha S, Ehlers SJ, Lee JY, Fernandez ML, Koo SI (2009). Dietary green tea extract lowers plasma and hepatic triglycerides and decreases the expression of sterol regulatory element-binding protein-1c mrna and its responsive genes in fructose-fed, ovariectomized rats. *Journal of Nutrition*.

[81] Wu LY, Juan CC, Ho LT, Hsu YP, Hwang LS (2004). Effect of green tea supplementation on insulin sensitivity in sprague-dawley rats. *Journal of Agricultural and Food Chemistry*.

[82] Nakamoto K, Takayama F, Mankura M (2009). Beneficial effects of fermented green tea extract in a rat model of non-alcoholic steatohepatitis. *Journal of Clinical Biochemistry and Nutrition*.

[83] Chung MY, Park HJ, Manautou JE, Koo SI, Bruno RS (2011). Green tea extract protects against nonalcoholic steatohepatitis in ob/ob mice by decreasing oxidative and nitrative stress responses induced by proinflammatory enzymes. *Journal of Nutritional Biochemistry*.

[84] Musso G, Gambino R, Pacini G, Pagano G, Durazzo M, Cassader M (2009). Transcription factor 7-like 2 polymorphism modulates glucose and lipid homeostasis, adipokine profile, and hepatocyte apoptosis in NASH. *Hepatology*.

[85] Ding X, Saxena NK, Lin S, Gupta N, Anania FA (2006). Exendin-4, a glucagon-like protein-1 (GLP-1) receptor agonist, reverses hepatic steatosis in ob/ob mice. *Hepatology*.

[86] Musso G, Gambino R, Pacini G, De Michieli F, Cassader M (2009). Prolonged saturated fat-induced, glucose-dependent insulinotropic polypeptide elevation is associated with adipokine imbalance and liver injury in nonalcoholic steatohepatitis: dysregulated enteroadipocyte axis as a novel feature of fatty liver. *American Journal of Clinical Nutrition*.

[87] Suckling K (2008). Selective thyromimetics for atherosclerosis and dyslipidaemia: another old target making progress. *Expert Opinion on Investigational Drugs*.

[88] Perra A, Simbula G, Simbula M (2008). Thyroid hormone (T3) and TR*β* agonist GC-1 inhibit/reverse nonalcoholic fatty liver in rats. *The FASEB Journal*.

[89] Zhou J, Febbraio M, Wada T (2008). Hepatic fatty acid transporter Cd36 is a common target of LXR, PXR, and PPAR*γ* in promoting steatosis. *Gastroenterology*.

[90] Marek CJ, Tucker SJ, Konstantinou DK (2005). Pregnenolone-16*α*-carbonitrile inhibits rodent liver fibrogenesis via PXR (pregnane X receptor)-dependent and PXR-independent mechanisms. *Biochemical Journal*.

[91] Ma K, Saha PK, Chan L, Moore DD (2006). Farnesoid X receptor is essential for normal glucose homeostasis. *Journal of Clinical Investigation*.

[92] Thomas C, Pellicciari R, Pruzanski M, Auwerx J, Schoonjans K (2008). Targeting bile-acid signalling for metabolic diseases. *Nature Reviews Drug Discovery*.

[93] Mummadi RR, Kasturi KS, Chennareddygari S, Sood GK (2008). Effect of bariatric surgery on nonalcoholic fatty liver disease: systematic review and meta-analysis. *Clinical Gastroenterology and Hepatology*.

[94] Luyckx FH, Desaive C, Thiry A (1998). Liver abnormalities in severely obese subjects: effect of drastic weight loss after gastroplasty. *International Journal of Obesity*.

[95] Georgescu EF, Ionescu R, Niculescu M, Mogoanta L, Vancica L (2009). Angiotensin-receptor blockers as therapy for mild-to-moderate hypertension-associated non-alcoholic steatohepatitis. *World Journal of Gastroenterology*.

[96] Vilar Gomez E, Rodriguez De Miranda A, Gra Oramas B (2009). Clinical trial: a nutritional supplement Viusid, in combination with diet and exercise, in patients with nonalcoholic fatty liver disease. *Alimentary Pharmacology and Therapeutics*.

